# The composition of the fluid phase in inclusions in synthetic HPHT diamonds grown in system Fe–Ni–Ti–C

**DOI:** 10.1038/s41598-022-05153-7

**Published:** 2022-01-24

**Authors:** Valeri Sonin, Anatoly Tomilenko, Egor Zhimulev, Taras Bul’bak, Aleksei Chepurov, Yuri Babich, Alla Logvinova, Tat’yana Timina, Anatoly Chepurov

**Affiliations:** grid.465281.c 0000 0004 0563 5291 V.S. Sobolev Institute of Geology and Mineralogy SB RAS, Koptyuga Ave. 3, Novosibirsk, Russia 630090

**Keywords:** Planetary science, Solid Earth sciences, Chemistry, Materials science

## Abstract

Diamonds grown by high pressure high temperature process (HPHT) are usually characterized by yellow color and high contents of nitrogen. Introduction of Ti decreases nitrogen content in diamond. Understanding the formation of nitrogen-poor diamond is very important not for the progress of HPHT process only, but because these diamond varieties represent the rare natural stones, although their crystallization conditions have not been clarified yet. Here we studied the composition of fluid phase in synthetic diamonds. The experiments were performed using a high-pressure apparatus BARS at pressures 5.5–6.0 GPa and temperatures 1350–1400 °C. It was found that introduction of metallic Ti leads to concentration of nitrogen mainly as nitrogenated hydrocarbons. The hypothesis that elucidates the formation of low-nitrogen diamond in Fe–Ni is proposed: the presence of Ti leads to an increase of hydrogen fugacity in the metal melt which drastically reduces the nitrogen solubility. As a result, nitrogen concentrates in the form of complex hydrocarbon compounds, while diamond grows colorless and characterized by very low nitrogen content. It is suggested that the proposed mechanism acts the same way in the presence of other metals which are strong reducing agents.

## Introduction

Fe-based melts are widely used for growing synthetic diamonds at high pressures and high temperatures. In order to decrease the melting point and suppress the process of carbide formation, Ni (or Co) is commonly added to Fe. Diamonds grown by this process are characterized by yellow color and high contents of nitrogen as a structural impurity, which reaches concentrations between 50–100 and 1000 ppm^1,2^. Nitrogen is captured by growing diamond crystal in the form of single atoms that is a result of nitrogen dissociation in melts of transition metals of Fe group. The most common source of nitrogen in HPHT process is the air that present in pores of high pressure cell during its assembly. At the temperatures of experiment higher than 1500 °C, single nitrogen atoms in diamond structure aggregate into pairs^3^. nitrogen solubility in Fe melt obeys the Sieverts’ law, i. e. at higher partial pressure of nitrogen its solubility in the melt increases^4^.

Titanium is the most effective additive to alloys that can increase the solubility of nitrogen in Fe and Ni melts^5,6^. However, a paradoxical situation is observed: the introduction of Ti into the growth system must increase the solubility of nitrogen in the Fe–Ni melt, but as a result the amount of nitrogen in diamond decreases. It is known that only a few percent of Ti in the system leads to growth of colorless diamond, in which nitrogen content does not exceed 1 ppm^3^. That is also confirmed by the experiments with higher Ti additives, which demonstrated a significant decrease of nitrogen impurity in diamond, respectively^7^. In commercial HPHT diamond growth, other IVB-elements are also used: Zr and Hf^8^, especially in the last decade according to LA-ICP-MS studies^9^. These elements are often called “nitrogen getters”.

At least two hypotheses exist in explaining the role of Ti for crystallization of low-nitrogen diamonds (types IIa and IIb according to the physical classification). The IVB elements are considered to bind nitrogen to nitrides^8^. It should be emphasized that nitrides are still not found in products after HPHT experiments. It is known that nitrides of Ti, Zr, and Hf are stable at high P–T parameters, but only in the nitrogen-rich medium^10^. The most popular explanation is that during experiment at high P–T the nitrides tend to form transition clusters^2^. The other hypothesis is based on the experimental fact of crystallization of nitrogen-rich diamond if Ti is added in the form of TiO_2_. The introduction of metallic Ti reduces oxygen concentration in the melt, because metallic Ti acts as a strong reducing agent: oxygen fugacity (ƒ_O2_) that corresponds to Ti-TiO_2_ equilibrium at 5.0 GPa and 1400 °C is 10 orders of magnitude lower than that of Fe-FeO^11^. Fe is reduced from Fe-bearing oxides and silicates in the presence of metallic Ti at high pressure and temperatures^12^. Therefore, it can be summarized that the mechanism of crystallization of low-nitrogen diamond in the presence of Ti has not been clarified yet.

Synthetic HPHT diamonds often contain inclusions of solid metal alloy as well as the other solid phases^8,13–16^. Carbide phases, including TiC, were also found in the experimental products^2,3,8^. The stability of TiC at high P–T is supported by its presence in Fe–Ti–C system after the diamond synthesis experiments^17^. Additionally, diamonds also contain the so-called “cloud-like” inclusions^9,13^. Understanding the formation conditions of low-nitrogen diamond is very important not for the progress of HPHT technology only. Nitrogen-poor colorless diamonds are much more valuable for use in jewelry than yellow diamonds containing nitrogen impurities in a non-aggregated form. In addition, nitrogen-poor diamonds are a unique raw material for electronic technology. It should also be noted that nitrogen-poor diamonds are very rare in nature. At many deposits, their occurrence is about 2%^18^. Moreover, most natural nitrogen-poor diamonds are plastically deformed^19^, which complicates their use as a semiconductor material. Synthetic nitrogen-poor diamonds are of a much higher quality. In this work we studied the composition of the fluid phase in synthetic HPHT diamonds with the aim to evaluate the state of nitrogen in diamond growth medium, and to elucidate the effect of Ti on the nitrogen state. The composition of inclusions, including fluid ones, is widely used to identify the crystallization conditions of synthetic diamonds^9,13^, and the study of inclusions in natural diamonds is the only way to evaluate the mineral composition of the upper and even the lower mantle of the Earth^20–22^.

## Results

**Figure 1:**
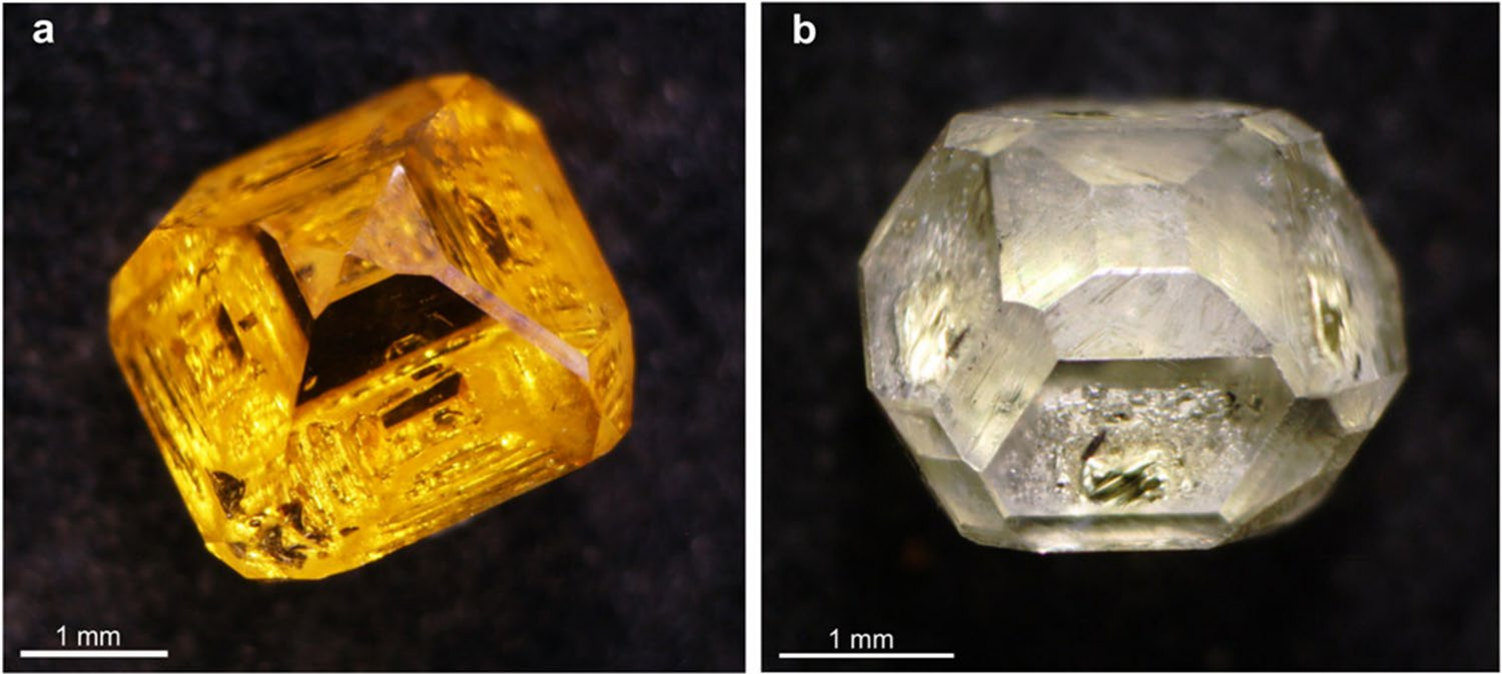
(**a**) Yellow synthetic diamond crystal grown in Fe–Ni–C system; (**b**) near-colorless synthetic diamond crystal grown in Fe–Ni–Ti–C system.

**Figure 2:**
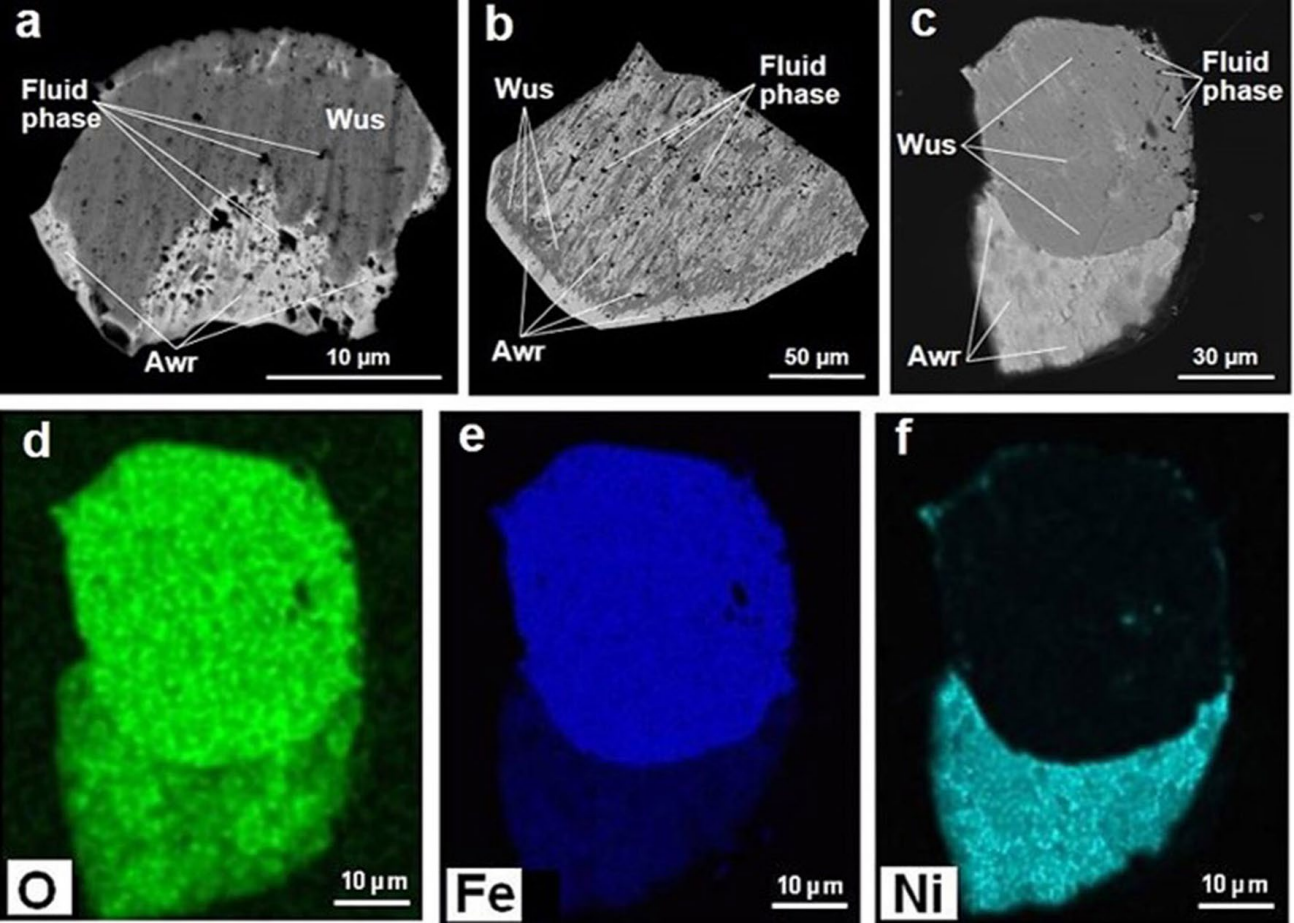
(**a–c**) BSE images of melt inclusions in synthetic diamonds grown in Fe–Ni–C system; (**d–f**) element maps for melt inclusion indicated on (**c**). *Wus* wustite, *Awr* awaruite.

Diamond crystals grown in Fe–Ni–C system are characterized by yellow color and octahedral habit with minor faces of {100}, {311}, {110} (Fig. 1a). The morphology of colorless diamonds grown in Fe–Ni–Ti–C system was a combination of faces {111} + {100} + {311} ± {511} + {110} (Fig. 1b). All melt inclusions in synthetic diamonds (Supplementary Fig. S1) grown in Fe–Ni–C system are completely crystallized and represented, mainly, by wustite (FeO) and awaruite (NiFe) (Fig. 2). In addition, the melt inclusions contain fluid segregations on the inner walls of which amorphous carbon was detected (Supplementary Fig. S2)^23^. The Raman spectroscopy data show that these fluid segregations contain methane, higher molecular weight hydrocarbons and hydrogen (Supplementary Fig. S3).

**Figure 3:**
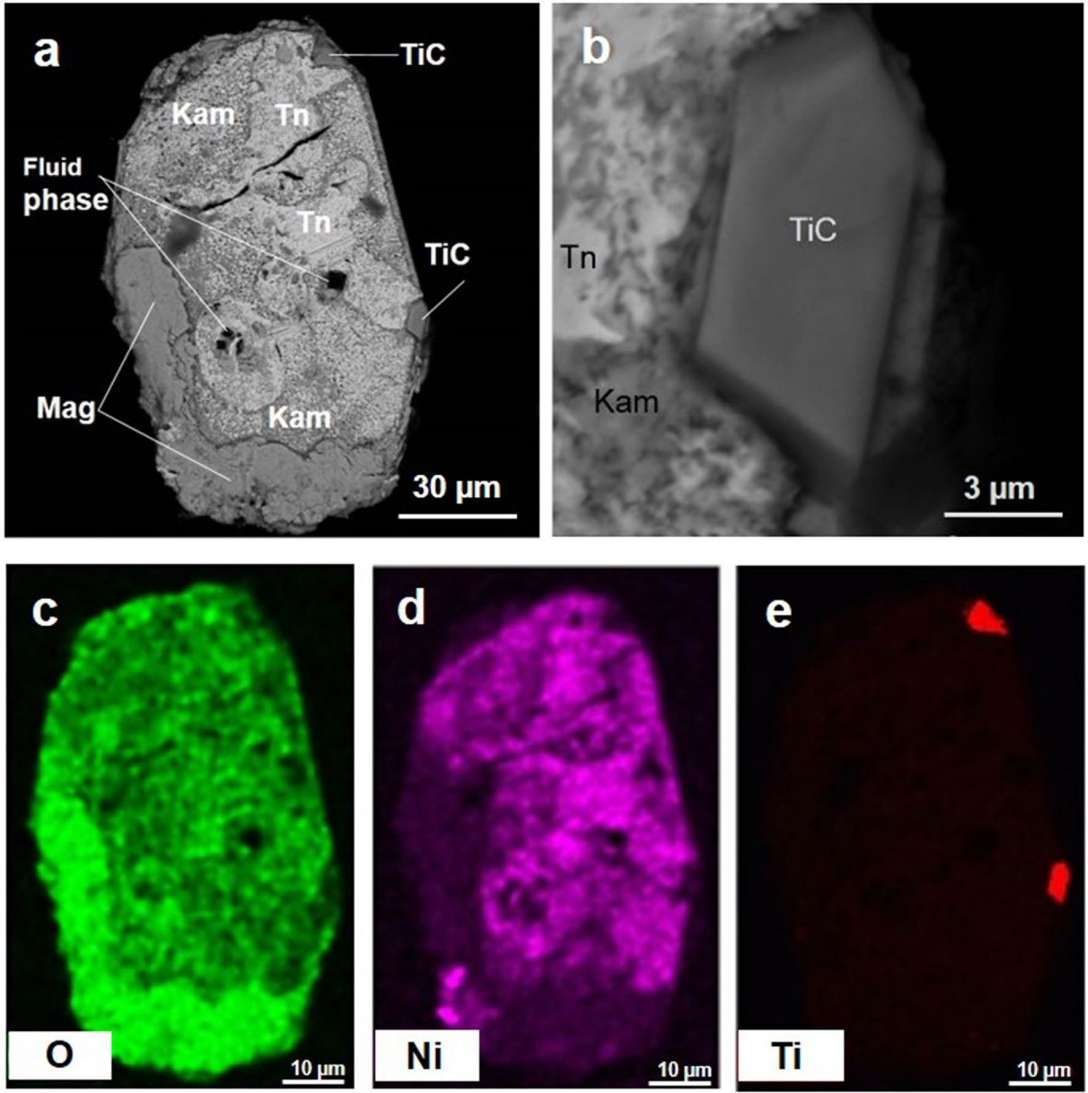
Melt inclusion in synthetic diamond from Fe–Ni–Ti–C system: BSE image of the common shape of inclusion shown on (**a**), and its enlarged fragment (**b**); (**c**–**e**) element maps for O, Ni and Ti. *Tn* taenite, *Kam* kamasite, *Mag* magnetite, *TiC* titanium carbide.

According to the data of optical and scanning electron microscopy, as well as Raman spectroscopy, melt inclusions in synthetic diamonds grown in Fe–Ni–Ti–C system, contain taenite (NiFe), kamacite (FeNi), a fluid phase and titanium carbide (TiC was determined on the basis of mass deficit in the microprobe analysis) (Fig. 3, Supplementary Fig. S4). It is worth noting that along with the FeNi alloy, melt inclusions also contain magnetite. The presence of magnetite is also proven by Raman spectroscopy (Supplementary Fig. S5). Magnetite is concentrated on the periphery of inclusions. This is probably due to the release of excess oxygen dissolved in Fe–Ni melt in the form of Fe_3_O_4_ during the sample cooling. The IR spectra of synthetic diamond crystals grown in the Fe–Ni–C and Fe–Ni–Ti–C systems are shown in Supplementary Fig. S6. It shows that synthetic diamond from Fe–Ni–C system has an IR spectrum with a pronounced absorption in the region of 900–1400 cm^−1^ that corresponds to nitrogen-centers C, N+ and A with predominance of nitrogen in a single form. The total content of nitrogen is 105–108 ppm (Supplementary Fig. S6, line 1). However, diamond grown in Fe–Ni–Ti–C system shows minimum absorption from nitrogen-centers and, respectively, a minimal content of single nitrogen no more than 3–4 ppm (Supplementary Fig. S6, line 2).

**Figure 4:**
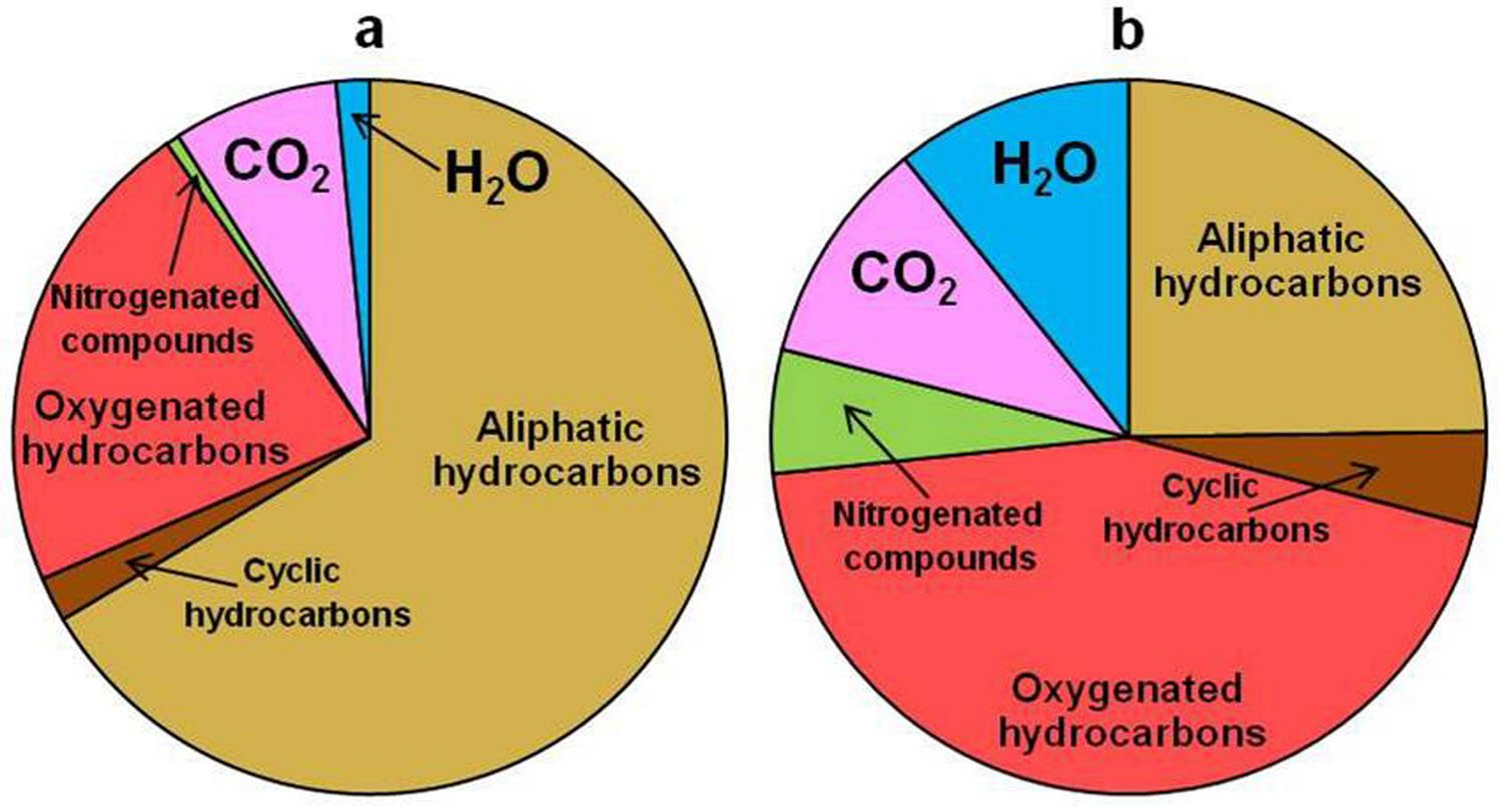
Relative contents of volatiles in synthetic diamonds grown in systems Fe–Ni–C (**a**) and Fe–Ni–Ti–C (**b**).

According to GC–MS analysis, the main volatile components released after mechanical crushing of diamonds are hydrocarbons and their derivatives: aliphatic (paraffins and olefins), cyclic (naphthenes and arenes), oxygenated (alcohols, ethers and esters, aldehydes, ketons, and carboxylic acids), heterocyclic compounds (dioxanes and furans), nitrogenated- and sulfonated compounds as well as carbon dioxide and water (Fig. 4, Supplementary Figs. S7–S9, Supplementary Table S1). The relative content of hydrocarbons and their derivatives in the diamonds grown in Fe–Ni–C and Fe–Ni–Ti–C systems is 90.1 and 83.3 rel%, respectively (Fig. 4, Supplementary Fig. S9, Supplementary Table S1). At the same time, the fraction of aliphatic hydrocarbons (65.6 rel%) is considerably higher in diamond grown in Fe–Ni–C system than that of Fe–Ni–Ti–C system (24.1 rel%). In contrast, the concentration of O-bearing hydrocarbons is significantly higher in diamond grown in Fe–Ni–Ti–C system compared to diamond from Fe–Ni–C system: 43.1 and 21.6 rel%, respectively. Additionally, the content of alcohols, ethers (to 17.2 rel%) and carboxylic acids (to 7.04 rel%) decreased considerably (Supplementary Table S1).

A drastic difference was also found in the amount of nitrogenated compounds detected in volatiles which were extracted from the diamonds. Nitrogen was identified both in a molecular form and in the form of nitrogenated hydrocarbon compounds (Supplementary Table S2). The content of molecular nitrogen in diamond from Fe–Ni–C system is 0.08 rel%, while in that from Fe–Ni–Ti–C system it equals 0.03 rel%. Diamond grown in Fe–Ni–C melt contains only one nitrogenated compound—acetonitrile in the amount of 0.52 rel%, whereas diamond from Fe–Ni–Ti–C is distinguished by the presence of 24 derivatives (from acetonitrile C_2_H_3_N to dodecanitrile C_13_H_23_N) (Supplementary Table S2). Their content is about 5.5 rel%. It is worth noting that nitrogen-poor natural and synthetic CVD diamonds also contain the elevated amounts of nitrogenated compounds^24–26^. Inclusions in diamonds from the both systems contain sulfonated compounds in the amount of 0.4 rel%, which are represented by sulfur dioxide (SO_2_), carbon disulfide (CS_2_), dimethyl disulfide (C_2_H_6_S_2_) and thiophenes (C_4_H_4_S–C_7_H_10_S). The content of H_2_O and CO_2_ in diamonds from the both systems is 1.5 versus 10.5 rel%, and 7.4 versus 10.0 rel%, respectively.

## Discussion

Thermodynamic modelling of C–H and C–H–O systems demonstrates that high-molecular hydrocarbons become stable at high pressure^27–29^. This phenomenon was recently confirmed by the experiments^30–32^. It should be noted that fluid inclusions, including high-molecular weight hydrocarbons, were found in synthetic and natural diamonds by the use of microthermometry and Raman spectroscopy^33–36^. However, a detailed identification of hydrocarbons and their derivatives in melt and fluid inclusions has became possible only recently as the GC–MS technique appeared^37^.

The results of GC–MS study demonstrated a complex composition of volatiles trapped in diamond crystals from Fe–Ni–C system^37^, while the main component is a group of aliphatic hydrocarbons (paraffins and olefins 47 and 18 rel%, respectively) (Supplementary Table S1). These diamonds are characterized by a low content of nitrogenated compounds (0.6 rel%), and IR spectroscopy shows that total content of structural nitrogen is 105–108 ppm. The introduction of metallic Ti into Fe–Ni–C system significantly changed the composition of volatiles (Supplementary Table S1): the amount of aliphatic hydrocarbons decreased considerably (down to 24 rel%), at the same time the fraction of oxygenated hydrocarbons increased drastically (up to 43 rel%). It is important to emphasize that with the presence of metallic Ti in the melt, the content (to 5.4 rel%) and the amount (to 24) of nitrogenated compounds increased significantly. And as the GC–MS analyses showed, crystallization of diamond occurred in a more oxidized environment (H/(O + H) value varies from 0.95 to 0.88; Supplementary Table S1).

The melting points of pure metals Fe and Ni are higher than the temperatures chosen for the diamond growth experiments at the same pressure^1,38–43^. Metallic Ti has even higher melting point^44–46^. In HPHTprocess the pressure is loaded first, and only then the sample is heated up to a required temperature. The melting starts on the contact of Fe–Ni with graphite (C-source) as a result of lower eutectic temperature compared to the melting point of pure metals. At this stage, an important condition is to overcome the so-called “carbide barrier”, i.e. the melting points of carbides Fe(± Ni)_3_C and Fe(± Ni)_7_C_3_^47^. After that moment the synthesis of diamond from graphite starts in C-source. Growth of diamond monocrystal on seeds occurs as a next stage due to recrystallization of diamond material from C-source to the seed diamond.

At the initial stage of experiment Ti remains in a solid state, but due to its high reactivity with oxygen, it forms an oxide that absorbs oxygen from the crystallization chamber. The formation of Ti_2_O and TiO at high P–T was proved by experiments^12^. It is proposed that an extremely high reactivity of metallic Ti is a key factor that leads to crystallization of low-nitrogen diamond. Also, that is the reason why introduction of Ti in the form of oxide with a maximum degree oxidation (TiO_2_) did not affect the N-state: metallic Ti extracts oxygen from Fe–Ni melt, but TiO_2_ does not^11^. Nevertheless, crystallization of low-nitrogen diamond is related rather to the behavior of hydrogen, than oxygen. It is known that a decrease of oxygen fugacity in the C–O–H system results in an increase of hydrogen fugacity and vice versa. Therefore, binding of oxygen into titanium oxides must increase the concentration of hydrogen in Fe–Ni melt. The solubility of both hydrogen and nitrogen in melts of transition metals Fe, Ni and Co obeys the Sieverts’ law^48^. It is also known that addition of metallic Ti to Fe-melt leads to a drastic increase in H solubility^49,50^.

As noted in the “Methods” section we did not introduce any hydrogen into the growth chamber, besides that present in the pores of assembly as H_2_O. In experiment H dissolves in Fe–Ni melt by the following reactions:

$${\text{Fe }} + {\text{ H}}_{{2}} {\text{O }} \to {\text{ FeO }} + {\text{ H}}_{{2}} ;$$

$${\text{Ni }} + {\text{H}}_{{2}} {\text{O }} \to {\text{ NiO }} + {\text{ H}}_{{2}} .$$

The concentration of oxygen in the growth environment is determined by the buffer equilibrium:

$${2}\left( {{\text{Fe}},{\text{Ni}}} \right) \, + {\text{ O}}_{{2}} = { 2}\left( {{\text{Fe}},{\text{Ni}}} \right){\text{O}}.$$

At high P–T Ti binds oxygen and, therefore, restores metallic Fe and Ni from FeO and NiO oxides, as followed from the fugacity values of O_2_ in equilibrium buffer reactions (Supplementary Fig. S10)^11,51^. On the one hand, the reactions increase hydrogen solubility in the melt. On the other hand, this effect leads to a deficit of hydrogen in the fluid phase. At the chosen parameters of experiment, different hydrocarbon compounds are thermodynamically stable in fluid, while the emerging deficit of hydrogen is compensated by nitrogen atoms which enter the hydrogen positions in hydrocarbons. As a result, nitrogen that was initially present in the form of dissociated atoms in Fe–Ni melt, becomes concentrated mainly in nitrogenated compounds. Therefore, the higher is the amount of added metallic Ti, the higher is the content of hydrogen dissolved in Fe–Ni melt at a nearly equal amount of initial oxygen in the growth medium. And, finally, the lower is the content of nitrogen in diamond^3,7,8^. In addition, close to solid C-source (diamond) the interaction with Ti oxides leads to the formation of Ti carbide by the following reactions:

$${\text{2Ti}}_{{2}} {\text{O }} + {\text{ 4C }} \to {\text{ 4TiC }} + {\text{ O}}_{{2}} ;$$

$${\text{2TiO }} + {\text{ C }} \to {\text{ TiC }} + {\text{ O}}_{{2}} .$$

As a result, oxygen is released into the melt locally, which is accompanied by the appearance of sparse iron oxide Fe_3_O_4_ after sample cooling (e.g. inclusions represented in Fig. 3, Supplementary Fig. S4), as well as the elevated content of oxygen-bearing hydrocarbons in the fluid (Supplementary Table S1).

It should be emphasized that in the diamond growing experiments the carbon content exceeds its solubility in the metal melt. It is known that carbide and nitride formation are competing processes. Since the content of carbon in the medium significantly exceeds that of the nitrogen the Ti–C reaction is a prevailing one. The known experiments support these estimates demonstrating the crystallization of diamond in Fe–C system highly enrich with nitrogen in the form of NaN_3_. It was found that with an increase in nitrogen content in the initial charge the amount of Fe_x_N both in the run products and inclusions in diamond increased due to a decrease in the amount of iron carbide^52^. It should be added that nitrogen content in common Fe–Ni–Ti–C experiments is very low since its only source is the surrounding air captured by the materials of high-pressure cell during the assembly.

Diamonds with a low nitrogen content can also be grown if Al or Mg are added to the system, although their effect is less pronounced^7,8,14^. These metals (the same as Ti, Zr and Hf) are also carbide-, nitride- and hydride-forming elements, but their main peculiarity is that they are strong reducing agents. It can be summarized, that the reason facilitating the growth of low-nitrogen diamond in the presence of Al and Mg is obviously the same as in case of Ti.

## Conclusions

This paper reports the study of the composition of fluid phase from melt inclusions in diamonds grown on a seed in Fe–Ni–C and Fe–Ni–C–Ti melts. On the one hand, it was shown that in diamond from Fe–Ni–C nitrogen is concentrated mainly in the form of impurity centers that makes diamond yellow colored. On the other hand, with the introduction of metallic Ti, nitrogen was identified mainly as nitrogenated hydrocarbon compounds, in which it occupies the position of hydrogen atom. The hypothesis that elucidates the formation of low-nitrogen near-colorless diamond in Fe–Ni melts is proposed: the presence of Ti in Fe–Ni–C leads to an increase of hydrogen fugacity in the metal melt, which, in turn, reduces the solubility of nitrogen. As a result, nitrogen concentrates in the form of complex hydrocarbon compounds in the fluid, while diamond grows colorless and characterized by very low nitrogen content. It should be added that diamonds with minor nitrogen impurity are also grown with addition of other metals such as Al or Mg, although the effect is less pronounced. These metals, as well as Ti, Zr, Hf are considered carbide-, nitride- and hydride-forming elements, although the main peculiarity is that they are strong reducing agents. Therefore, it is suggested that the proposed mechanism of formation of low-nitrogen diamond in the presence of other metals such as Al, Mg, Zr, Hf^2,3,7,8,14^ or even rare earth elements (REE)^53^ is the same as in the case of Ti.

## Methods

### HPHT experiment

The experiments were performed using a multi-anvil high-pressure apparatus of the split-sphere type (BARS) at the Institute of Geology and Mineralogy of the Siberian Branch of the Russian Academy of Sciences in Novosibirsk according to the state assignment. Container made from ZrO_2_ had a rectangular parallelepiped shape. Its size was 20 × 20 × 23 mm with truncated edges and vertices. The tube-shaped heater was made of graphite. Molybdenum discs were used as electrical contacts. Pressure in the cell was calibrated at room temperature using the reference substances Bi and PbSe^54^. Temperature was measured with a PtRh_30_–PtRh_6_ thermocouple without a pressure correction. The temperature correction for pressure was determined by the melting curve of pure Ag and Au^55^. The experiments were carried out at pressures 5.5–6.0 GPa and temperatures 1350–1400 °C with duration over 45 h in the diamond stability field^56^. The details of the procedure are described in^11,15,57^. The measurement errors were ± 0.2 GPa and ± 25 °C. The samples were heated after pressurization. At the end of experiment the samples were cooled by quenching, which was 2–3 from the run temperature to 1000 °C. The crystallization chamber of the cell is a cylindrical capsule made from MgO. The assembly consisted of a source of carbon (graphite) and a catalyst metal (Fe_64_Ni_36_). Synthetic diamond microcrystals of 0.5 mm in size were used as seed crystals. Graphite was placed in the higher temperature zone of the assembly in order to provide temperature gradient between the carbon source and the seeds^58^. To obtain diamond crystals of type IIa, metallic Ti (7.6 wt% of the weight of Fe_64_Ni_36_) was added to the assembly. It should be added that the sample was assembled at room conditions, therefore air (O_2_, CO_2_, N_2_, H_2_O) was present in the pores of materials. The schematic drawing of high pressure cell assembly is shown in Supplementary Fig. S11.

### Fourier-transform infrared spectroscopy analysis

Infrared absorption spectra were recorded on a Bruker Vertex 70 FTIR spectrometer equipped with a HYPERION 2000 IR microscope using a square aperture 100 × 100 µm in size in the region 600–7500 cm^−1^; spectral resolution, 1 cm^−1^) performed in the V.S. Sobolev Institute of Geology and Mineralogy, Siberian Branch of the Russian Academy of Sciences, Novosibirsk. The background was corrected, and the spectra were normalized to the internal standard using the absorption coefficient (12.8 cm^−1^) of two-phonon spectrum of the diamond crystalline lattice at 2030 cm^−1^^18^. The spectra were analyzed using a specialized software IR’nDi-Module for processing the IR spectra of diamonds with decomposition into nitrogen components of synthetic diamonds: C-defects (single substituting nitrogen atoms), A-defects (two nitrogen atoms in adjacent substituting positions) and N+ defects (single substituting nitrogen atoms in the charge state + 1)^59^.

### Raman spectroscopy analysis

The compositions of gas and crystal phases in melt and fluid inclusions were analyzed using Raman spectroscopy^23,60^ on a Horiba Lab Ram HR 800 spectrometer in the Institute of Geology and Mineralogy SB RAS, Novosibirsk. The Raman signal was excited using a solid state Nd YAG laser with a wavelength 532 nm and power 75 mW. Spectrum registration was carried out using a semiconductor Endor detector with Peltier cooling. To locate the point in the analyzed sample, a confocal spectrometer based on the OLYMPUS BX-41microscope with a 100× lens with a large numerical aperture was used. The analysis was performed in a backscattering geometry. The time of signal accumulation and the size of confocal aperture varied depending on the size of the analyzed phase. The minimum size of confocal aperture was 30 nm (for objects with a size of 5–10 µm), and the maximum size was 300 nm (for objects larger than 100 µm). The spectra were obtained in the range of 100–4200 cm^−1^. The time of signal accumulation ranged from 25 s/spectral window for large objects and to 400 s/spectral window for small objects. The error of determination was within 1 cm^−1^. The Origin 8 software package was used for working with Raman spectra**.**

### SEM analysis

Metal melt inclusions in synthetic diamonds grown in Fe–Ni–C and Fe–Ni–Ti–C systems were studied using the optical microscopy (MC2-Zoom, Olympus BX35), scanning electron microscope MIRA 3 LMU (TESCAN Orsay Holding) equipped with an INCA Energy 450 + Xmax-80 microanalysis system (Oxford Instruments Nanoanalisys Ltd.) and JEOL JXA-8100 microanalyzer in the Analytical Center IGM SB RAS.

### GC–MS analysis

Volatiles from the sample were analyzed using the coupled gas chromatography–mass spectrometry (GC–MS) method on a Focus GC/DSQ II Series Single Quadrupole MS analyzer (Thermo Scientific, USA) at the V.S. Sobolev Institute of Geology and Mineralogy, Siberian Branch of the Russian Academy of Sciences, Novosibirsk^24,25,30,61–63^. The gas mixture was released from the fluid and melt inclusions of the samples by means of shock mechanical crushing in a custom designed crusher (Supplementary Fig. S12). The crusher was heated to 160 °C and flushed with He to remove adsorbed volatiles. The released mixture was entrained in a He stream, without cryogenic focusing. Each analytical run was preceded and followed by blank analyses, which later were used in data processing. The gas mixture was injected into the analytical column of the GC–MS instrument through a 6-port 2-position Valco (USA) valve thermostated at 290 °C, at a constant He flow rate of 1.7 mL min^−1^, using vacuum compensation. The GC–MS transferline temperature was held at 300 °C. The gas mixture was separated in a Restek Rt-Q-BOND capillary column (100% divinylbenzene used as a stationary phase; length, 30 m; inner diameter, 0.32 mm; film thickness, 10 µm). The temperature program of the GC separation comprised an isothermal stage (70 °C for 2 min) followed by two heating ramps (25 °C min^−1^ to 150 °C and 5 °C min^−1^ to 290 °C), followed by the final isothermal stage at 290 °C for 100 min. Total ion current (TIC) electron ionization spectra were collected on a quadrupole mass-selective detector in the full scan mode at anelectron energy and emission current of 70 eV and 100 µA, respectively. Other experimental parameters were as follows: ion source T = 200 °C; multiplier voltage 1500 V; positive ion detection; the mass range 5 to 500 amu; scan rate 1 s^−1^; and scan rate 506.6 amu s^−1^. The start time of the analysis was synchronized with shock crushing of the samples.

The procedure for preparing the sample for analysis excluded its contact with any solvents and other possible contamination. The input of the mixture extracted from the sample during the shock crushing was carried out online in the He flow without concentration including cryofocus. This method does not pyrolyze the sample but heats it only in order to convert any water within the sample into a gas phase. In this case, it is the gas mixture that is analyzed in situ rather than pyrolyzate, which contains more oxidized compounds (H_2_O, CO, CO_2_, etc.) due to the reactions between the gas mixture compounds, the gas mixture and accumulator surface, and the gas phase compounds and the sample. Blank online analyses were carried out before and after the ‘‘working” analysis. The previous analysis made it possible to control the release of gases adsorbed by the sample surface, including atmospheric components, and to record the system blank at the end of the process. The degree and completeness of hydrocarbon and polycyclic aromatic hydrocarbon elution from the analytical column during temperature programming in a chromatograph thermostat were determined using the results of subsequent analysis. If necessary, the analytical column was thermoconditioned to achieve the required blank. The collected spectra were interpreted using both the AMDIS 2.73 (Automated Mass Spectral Deconvolution and Identification System) software and manually, with background correction against spectra from the NIST2020 and Wiley Registry 12th Edition Mass Spectral libraries (NIST MS Search 2.4). Peak areas in TIC chromatograms were determined using the ICIS algorithm Xcalibur (1.4SR1 Qual Browser). This method is suitable for the detection of trace volatile concentrations exceeding tens of femtograms. The relative concentrations (rel%) of volatile components in the studied mixture were obtained by normalizing the areas of individual chromatographic peaks to the total area of all peaks. The reliability of this normalization method was verified using external standards. Namely, certified Scotty Inc. NL 34522-PI and 34525-PI gas standards of methane–hexane alkanes were injected into the gas stream in the splitless mode by means of a volumetric gas-tight syringe or a special valve with replaceable loops for volumes ranging from 2 to 500 µL. The calibration quality was assessed using the coefficients of determination R^2^ of the relationships between the peak area and the injected amount. The respective R^2^ values were as follows: 0.9975 (16 *m/z*, n = 22) for methane, 0.9963 (26 + 30 *m/z*, n = 16) for ethane, 0.9986 (29 + 43 *m/z*, n = 15) for propane, 0.9994 (29 + 43 *m/z*, n = 17) for butane, 0.9935 (43 + 72 *m/z*, n = 6) for pentane, and 0.9909 (57 + 86 *m/z*, n = 5) for hexane. The concentration ranges of alkanes during calibration were similar to concentrations encountered in the experiments. The relative analytical uncertainty for C_1_–C_6_ alkane determination was below 5% (2σ)^61^.

## Supplementary Information

Supplementary Information.
