# Supplementary material for: The composition of the fluid phase in inclusions in synthetic HPHT diamonds grown in system Fe–Ni–Ti–C
Source: Sci Rep. 2022 Jan 24;12:1246. doi: 10.1038/s41598-022-05153-7 (PMC8786936; doi:10.1038/s41598-022-05153-7)
Supplement: Supplementary file 1 — Supplementary Information. [file 41598_2022_5153_MOESM1_ESM.pdf]

Supplementary information

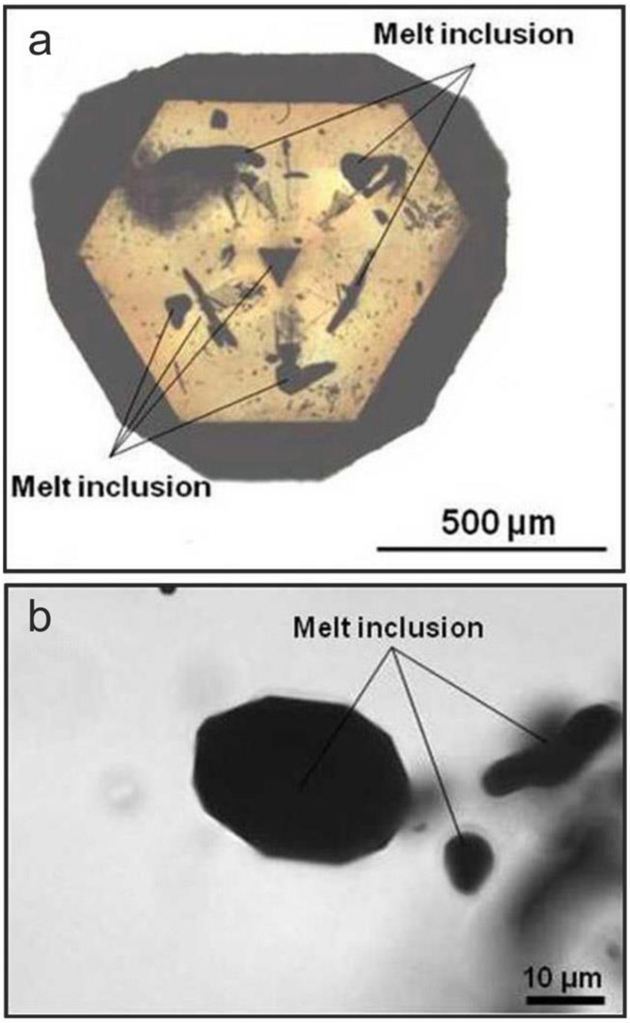

Supplementary Figure S1. (a) An example of diamond with melt inclusions (transmitted light), and (b) detailed view of the inclusions in diamond.

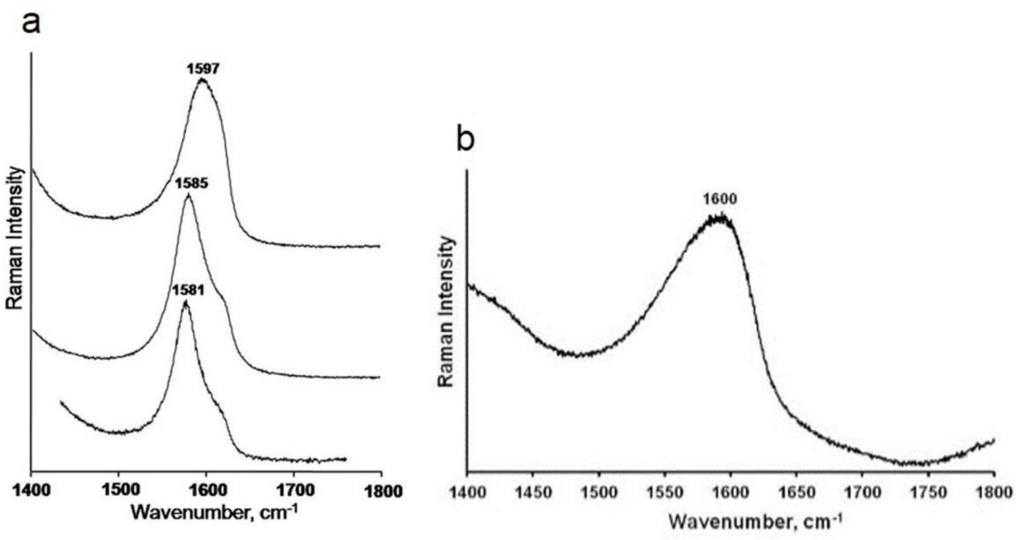

Supplementary Figure S2. Raman spectra of melt inclusion in synthetic diamond grown in Fe-Ni-C system: (a) graphite, lines 1581, 1585, 1587 cm^{-1}; (b) amorphous carbon, line 1600 cm^{-1} [23].

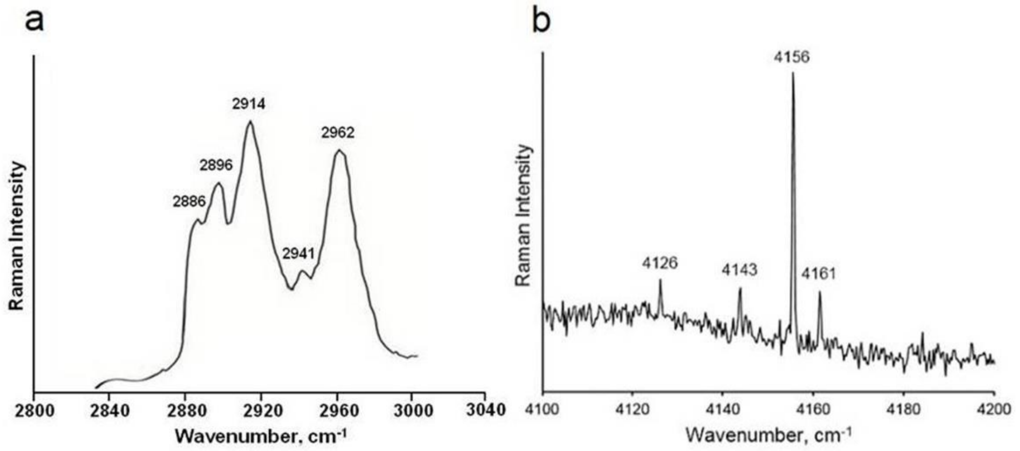

Supplementary Figure S3. Raman spectra of fluid phase included in synthetic diamond grown in Fe-Ni-C system: (a) methane, line 2914 cm^{-1} and heavier hydrocarbons, lines 2886, 2896, 2941, 2962 cm^{-1}; (b) hydrogen, lines 4126, 4143, 4156, 4161 cm^{-1} [23].

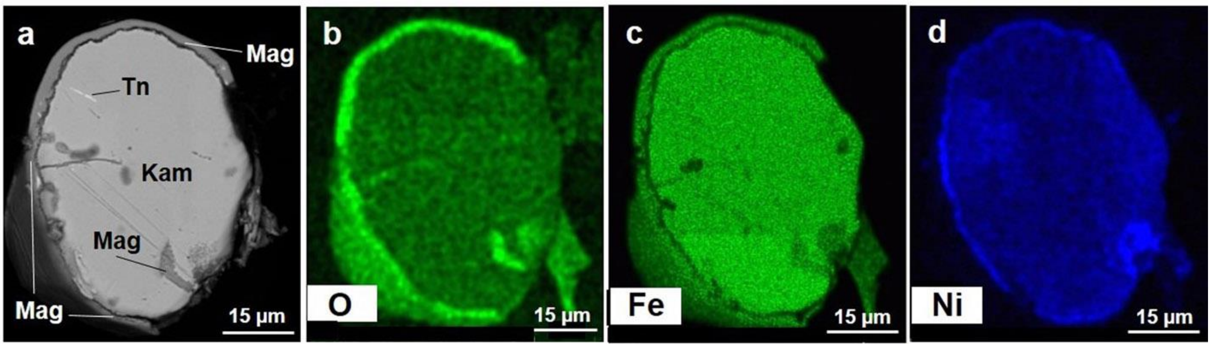

Supplementary Figure S4. Melt inclusion in synthetic diamond grown in Fe-Ni-Ti-C system: (a) BSE image; (b-d) element maps for O, Fe and Ni. Tn - taenite; Kam - kamacite; Mag – magnetite.

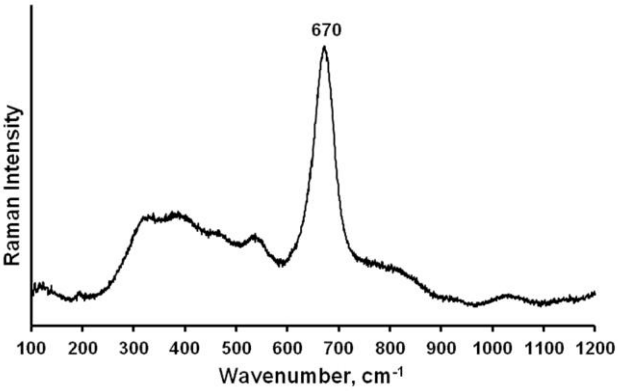

Supplementary Figure S5. Raman spectra of metal melt inclusion in synthetic diamond from Fe-Ni-Ti-C system, line 670 cm^{-1} corresponds to magnetite [23].

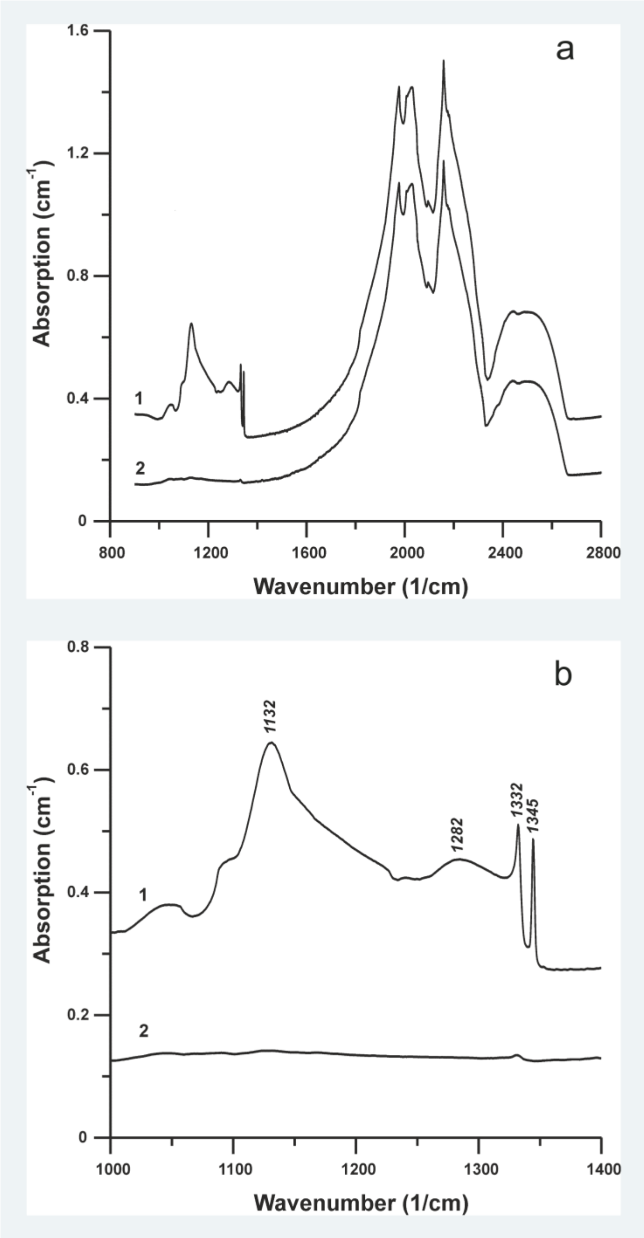

Supplementary Figure S6. IR-spectra overview (a) and enlarged one-phonon region (b) for two diamond samples (spectra are not normalized and shifted vertically for clarity): 1 – diamond grown in Fe-Ni-C system has a pronounced absorption in the 900-1400 cm^{-1} range due to nitrogen in form of C (90.8 ppm, main peaks at 1132 and 1345 cm^{-1}), N+ (8.4 ppm, main peak at 1332 cm^{-1}) and A (9.3 ppm, main peak at 1282 cm^{-1}); total nitrogen content 108 ppm. 2 – diamond grown in Fe-Ni-Ti-C system demonstrates low absorption in one-phonon region, total nitrogen content is lower than 3-4 ppm.

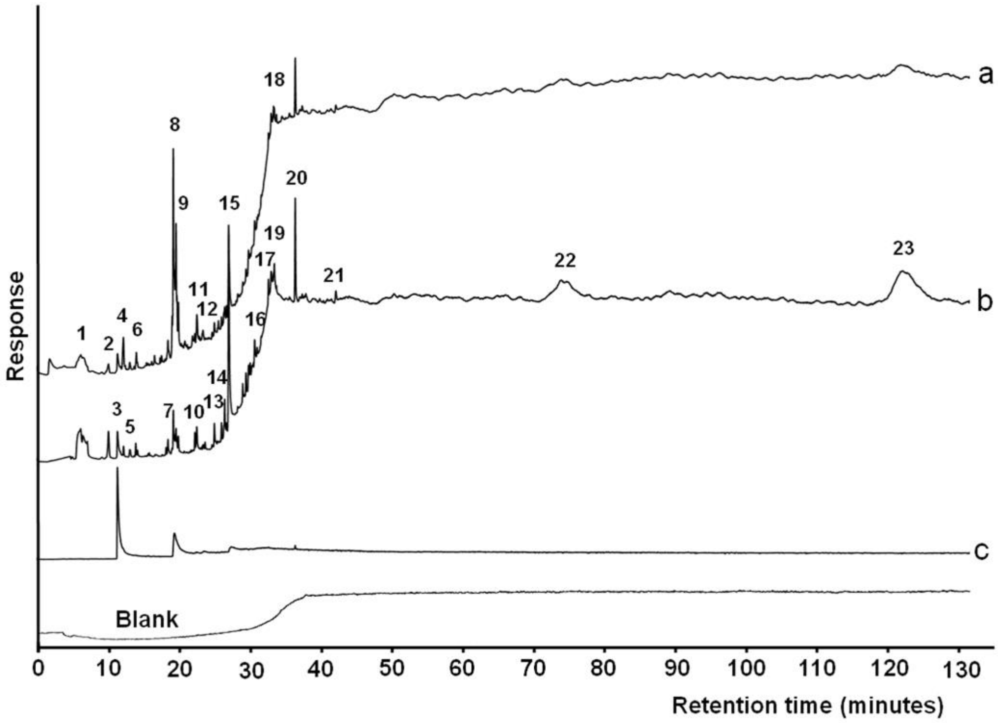

Supplementary Figure S7. Chromatograms of volatile components released after mechanical crushing of synthetic diamond grown in Fe-Ni-C system: (a) total ion chromatogram (TIC); (b) RIC (reconstructed ion chromatogram) *m/z* (43+57+71+85) (magnified 1x5) and (c) RIC *m/z* 60 (magnified 1x30). 1 – Acetonitrile (C_2H_3N); 2 – 2-Propanone (C_3H_6O); 3 – Acetic acid ($C_2H_4O_2$); 4 - Benzole (C_6H_6); 5 – 1,4-Dioxane ($C_4H_8O_2$); 6 – Methyl methacrylate ($C_5H_8O_2$); 7 – 4-Methyl-3-heptene (C_8H_{16}); 8 – (E)-4-Octene (C_8H_{16}); 9 - (E)-3-Octene (C_8H_{16}); 10 - (E)-2-Octene (C_8H_{16}); 11 – 2-Heptanone ($C_7H_{14}O$); 12 – 1,3,5-Trimethyl-1H-pyrazole ($C_6H_{10}N_2$); 13 – 2-Ethylhexanal ($C_7H_{14}O$); 14 – н-Octanal ($C_8H_{16}O$); 15 – 3-(Chloromethyl)-heptane ($C_6H_{13}Cl$); 16 – (E)-2-Methyl-3-udecene ($C_{12}H_{24}$); 17 – 2-Decanone ($C_{10}H_{20}O$); 18 – н-Decanal ($C_{10}H_{20}O$); 19 – 4-Dodecene ($C_{12}H_{24}$); 20 – 2-Undecanone ($C_{11}H_{22}O$); 21 – 2-Dodecanone ($C_{12}H_{24}O$); 22 – н-Heptadecane ($C_{17}H_{36}$); 23 – н-Nonadecane ($C_{19}H_{40}$).

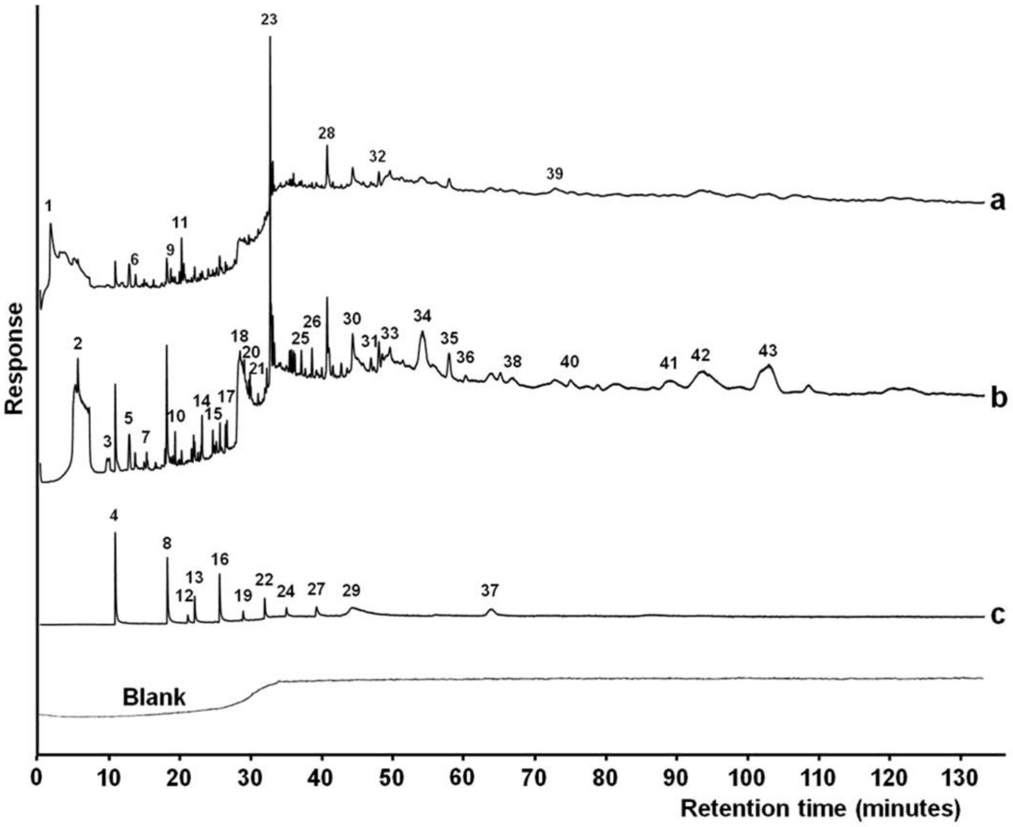

Supplementary Figure S8. Chromatograms of volatile components released after mechanical crushing of synthetic diamond grown in Fe-Ni-C-Ti system: (a) TIC (1x0.05); (b) RIC *m/z* (43+57+71+85) (1x0.5) and (c) RIC *m/z* 60. 1. Carbon dioxide (CO_2); 2. Butane (C_4H_{10}); 3. 2-Butanone (C_4H_8O); 4. Acetic acid ($C_2H_4O_2$); 5. 1,4-Dioxane ($C_4H_8O_2$); 6. 1-Heptene (C_7H_{14}); 7. Heptane (C_7H_{16}); 8. Butanoic acid ($C_4H_8O_2$); 9. 3-Methyleneheptane (C_8H_{16}); 10. 2-Octene (C_8H_{16}); 11. p-Xylene (C_8H_{10}); 12. 3-Methylbutanoic acid ($C_5H_{10}O_2$); 13. Pentanoic acid ($C_4H_8O_2$); 14. Nonane (C_9H_{20}); 15. 2-Ethylhexanal ($C_8H_{16}O$); 16. Hexanoic acid ($C_6H_{12}O_2$); 17. Decane ($C_{10}H_{22}$); 18. 2-Ethyl-1-hexanol ($C_8H_{18}O$); 19. Heptanoic acid ($C_7H_{14}O_2$); 20. Undecane ($C_{11}H_{24}$); 21. Pentylbenzene ($C_{11}H_{16}$); 22. Octanoic acid ($C_8H_{16}O_2$); 23. 1-Dodecene ($C_{12}H_{24}$); 24. Nonanoic acid ($C_9H_{18}O_2$); 25. γ-Nonanolactone ($C_9H_{16}O_2$); 26. Dipentyl ester carbonic acid ($C_{11}H_{22}O_3$); 27. Decanoic acid ($C_{10}H_{20}O_2$); 28. 1-Tetradecene ($C_{14}H_{28}$); 29. Undecanoic acid ($C_{11}H_{22}O_2$); 30. 1-Chlorododecane ($C_{12}H_{25}Cl$); 31. 2-Tridecanone ($C_{13}H_{26}O$); 32. 1-Pentadecene ($C_{15}H_{30}$); 33. 1-Tridecanol ($C_{13}H_{28}O$); 34. 3-Methylpentadecane ($C_{16}H_{34}$); 35. Dodecyl ester acetic acid ($C_{14}H_{28}O_2$); 36. 1-Hexadecene ($C_{16}H_{32}$); 37. Tridecanoic acid ($C_{13}H_{26}O_2$); 38. Chlorotetradecane ($C_{14}H_{29}Cl$); 39. Dipropyl phthalate ($C_{14}H_{28}O_4$); 40. 2-Pentadecanone ($C_{15}H_{30}O$); 41. 2-Hexadecanone ($C_{16}H_{32}O$); 42. Hexadecanal ($C_{16}H_{32}O$); 43. Octadecane ($C_{18}H_{38}$).

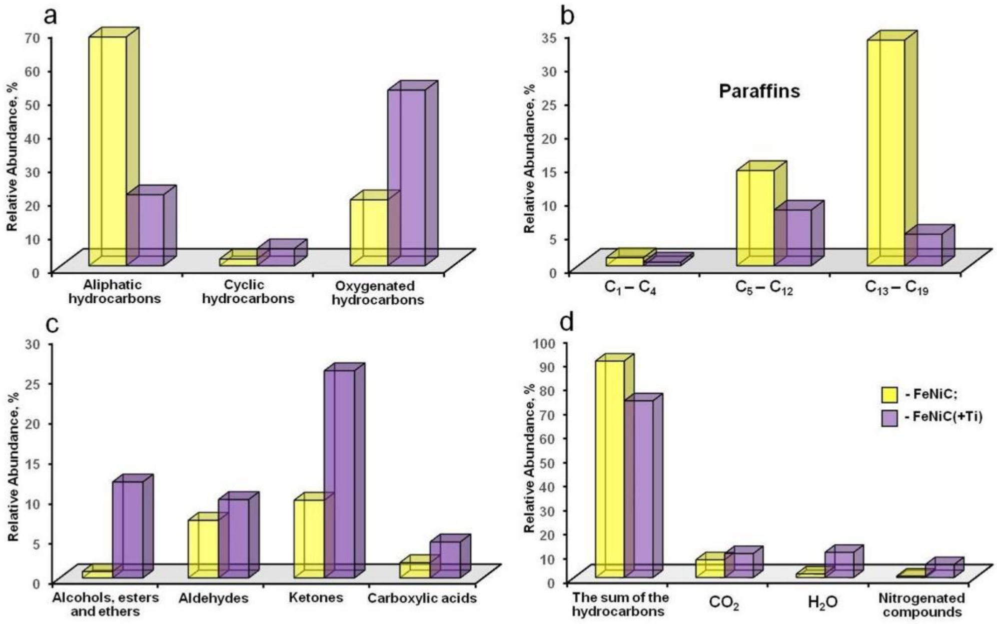

Supplementary Figure S9. Relative contents of volatile components in synthetic diamonds grown in Fe-Ni-C and Fe-Ni-Ti-C systems: (a) aliphatic, cyclic and oxygenated hydrocarbons; (b) «light» (C_1-C_4), «medium» (C_5-C_{12}) and «heavy» (C_{13}-C_{19}) hydrocarbons (paraffins); (c) alcohols, ethers and esters, aldehydes, ketones, carboxylic acids; (d) total amount of aliphatic, cyclic and oxygenated hydrocarbons, CO_2, H_2O and nitrogenated compounds.

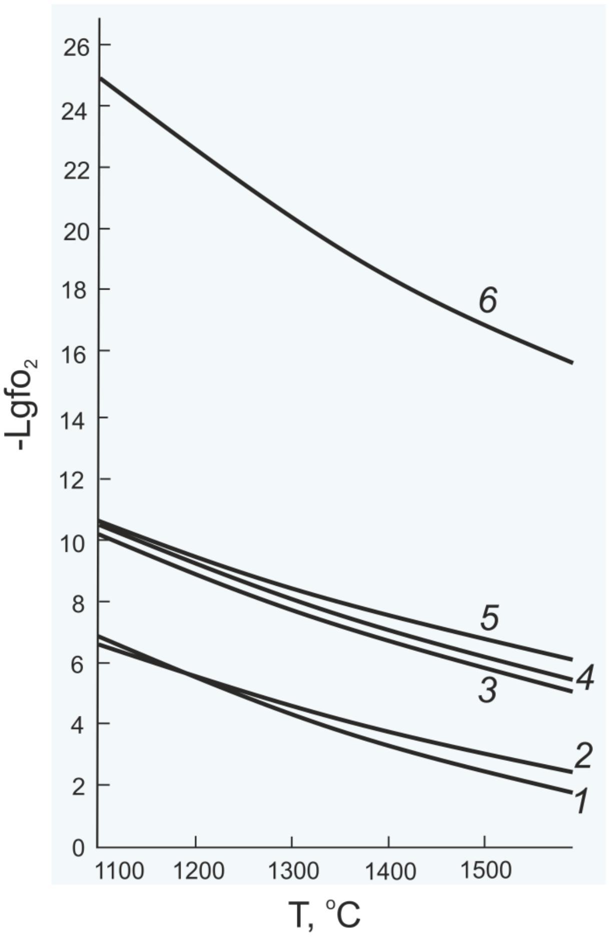

Supplementary Figure S10. Oxygen fugacity of buffer equilibriums at pressures 5.0 (lines 1-5) and 6.0 GPa (line 6) by [51,11]: 1 – FeO + Fe_3O_4; 2 – Ni + NiO; 3 – ($Fe_{0.7}Ni_{0.3}$, C, O)-melt + diamond + FeO; 4 – (Fe, C, O)-melt + diamond + FeO; 5 – Fe + FeO; 6 – Ti + TiO_2.

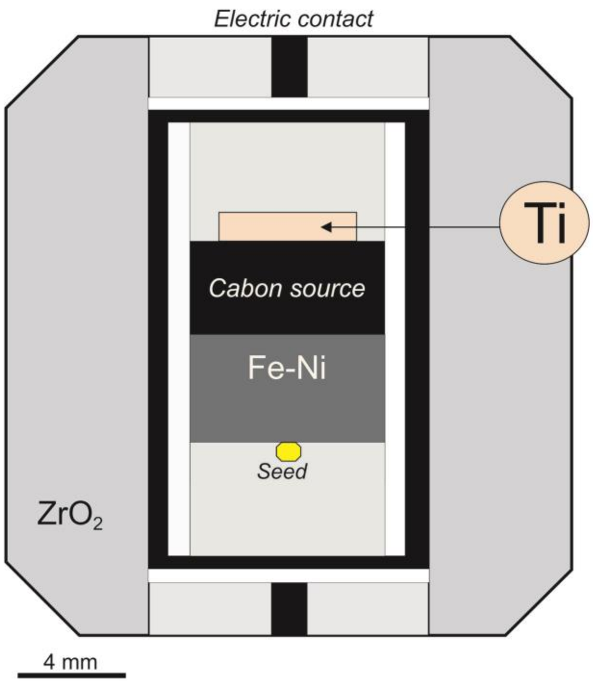

Supplementary Figure S11. Vertical cross-section of high pressure cell assembly used in experiments on BARS apparatus. Ti source is placed at the top of reaction chamber. Diamond crystal grows on seed in the bottom zone.

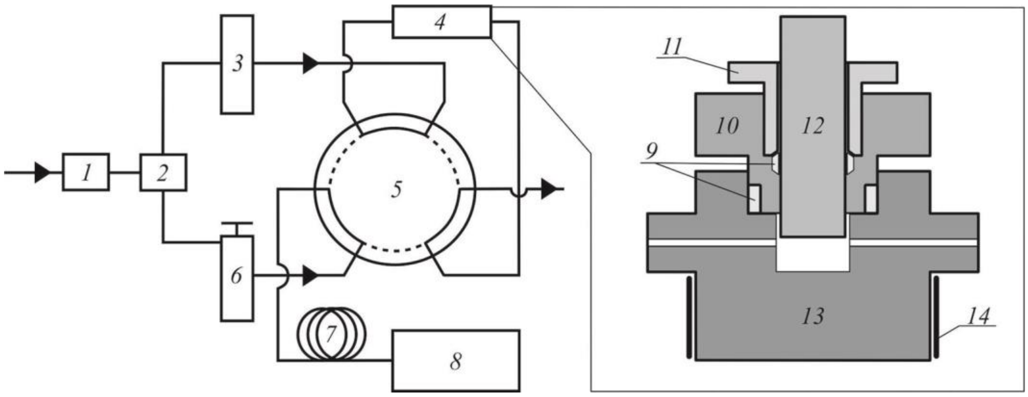

Supplementary Figure S12. Schematic diagram of the pneumatic circuit with the main nodes of the gas chromatograph-mass spectrometer used, by [25]: 1 - filter; 2 - tee; 3 - gas flow regulator; 4 - cell for sample destruction; 5 - 6-port 2-way valve; 6 - SSL injector; 7 - analytical column; 8 - mass selective detector. On the insert, there is a simplified section diagram of 4 nodes: 9 - fluoroplastic seals; 10 - persistent nut; 11 - pressure nut; 12 - shockproof stock; 13 - housing with a recess for the sample and a through gas path; 14 - furnace heater.

Supplementary Table S1. Composition of volatiles released after mechanical crushing of synthetic diamonds grown in metal-C systems (GC-MS data), (rel.%).

Component	Nominal mass	Fe-Ni-C	Fe-Ni-Ti-C
**Aliphatic hydrocarbons:**		**65.6**	**24.1**
Paraffins (CH_4-$C_{19}H_{40}$)	16-268	47.2	11.7
Olefins (C_2H_2-$C_{18}H_{36}$)	26-252	18.4	12.4
**Cyclic hydrocarbons:**		**2.0**	**4.16**
Cycloalkanes (naphthenes) (C_5H_{10}-$C_{15}H_{30}$)	70-210	0.4	1.93
Arenes (C_6H_6-$C_{15}H_{24}$)	78-204	1.59	2.21
Polycyclic aromatic hydrocarbons ($C_{10}H_8$-$C_{11}H_{10}$)	128-142	0.01	0.02
**Oxygenated hydrocarbons:**		**21.6**	**43.18**
Alcohols, esters and ethers (C_6H_6O-	94-306	0.7	17.2
$C_{18}H_{26}O_4$)	44-240	7.4	9.40
Aldehydes (C_2H_6O-$C_{16}H_{32}O$)	58-240	11.6	9.54
Ketones (C_3H_6O- $C_{16}H_{32}O$)	60-228	1.9	7.04
Carboxylic acids ($C_2H_4O_2$-$C_{14}H_{28}O_2$)			
**Heterocyclic compounds:**		**0.9**	**2.11**
Dioxanes ($C_4H_8O_2$)	88	0.4	1.29
Furans (C_4H_6O-$C_{13}H_{22}O$)	70-192	0.5	0.82
**Nitrogen** (N_2)	28	**0.1**	**0.1**
**Nitrogenated compounds** (NH_3-$C_{12}H_{23}N$)	17-181	**0.5**	**5.43**
**Sulfonated compounds** (SO_2-$C_{11}H_{24}S$)	64-188	**0.4**	**0.42**
**CO_2**	44	**7.4**	**10.0**
**H_2O**	18	**1.5**	**10.5**
**The number of identified components**		**104**	**194**
H/(O+H)		***0.95***	***0.87***
Alkanes/Alkenes		***2.60***	***0.94***

Supplementary Table S2. Composition of nitrogenated volatile compounds identified after mechanical crushing of synthetic diamonds grown in metal-C systems (GC-MS data).

Formula	Component	Fe-Ni-C	Fe-Ni-Ti-C
N_2	Nitrogen	+	+
C_2H_3N	Acetonitrile	+	+
C_2H_5NO	Acetamide	-	+
C_4H_7N	Butanenitrile	-	+
$C_4H_4N_2$	1,3-Diazine	-	+
C_4H_9N	Pyrrolidine	-	+
C_4H_9NO	Butanamide	-	+
C_5H_5N	Pyridine	-	+
$C_2H_5NO_2$	Nitroethane	-	+
$C_5H_8N_2$	1,4-Dimethylpyrazole	-	+
C_5H_9N	Pentanenitrile	-	+
C_6H_7N	3-Methylpyridine	-	+
$C_5H_{11}NO$	N-Isopropylacetamide	-	+
$C_6H_{11}N$	Hexanenitrile	-	+
$C_6H_{15}N$	N,N-Dimethyl-2-butanamine	-	+
C_7H_5N	Benzonitrile	-	+
$C_7H_{13}N$	4,4-Dimethylpentanenitrile	-	+
$C_7H_{13}N$	Heptanenitrile	-	+
$C_5H_9NO_2$	Nitrocyclopentane	-	+
$C_8H_{15}N$	1-Butyl-1H-pyrrole	-	+
$C_8H_{15}N$	Octanenitrile	-	+
C_8H_7N	Benzeneacetonitrile	-	+
$C_9H_{17}N$	Nonanenitrile	-	+
$C_{10}H_{19}N$	Decanenitrile	-	+
$C_{12}H_{23}N$	Dodecanenitrile	-	+
